# Resilience of Phytoplankton and Microzooplankton Communities under Ocean Alkalinity Enhancement in the Oligotrophic Ocean

**DOI:** 10.1021/acs.est.4c09838

**Published:** 2024-11-11

**Authors:** Xiaoke Xin, Silvan Urs Goldenberg, Jan Taucher, Annegret Stuhr, Javier Arístegui, Ulf Riebesell

**Affiliations:** † GEOMAR Helmholtz Centre for Ocean Research, Wischhofstraße 1-3, Kiel 24148, Germany; ‡ Instituto de Oceanografía y Cambio Global (IOCAG), Universidad de Las Palmas de Gran Canaria, Parque Científico Tecnológico Marino de Taliarte, Telde 35214 Las Palmas, Spain

**Keywords:** carbon dioxide removal, carbonate chemistry, plankton response, community composition, ecological impacts

## Abstract

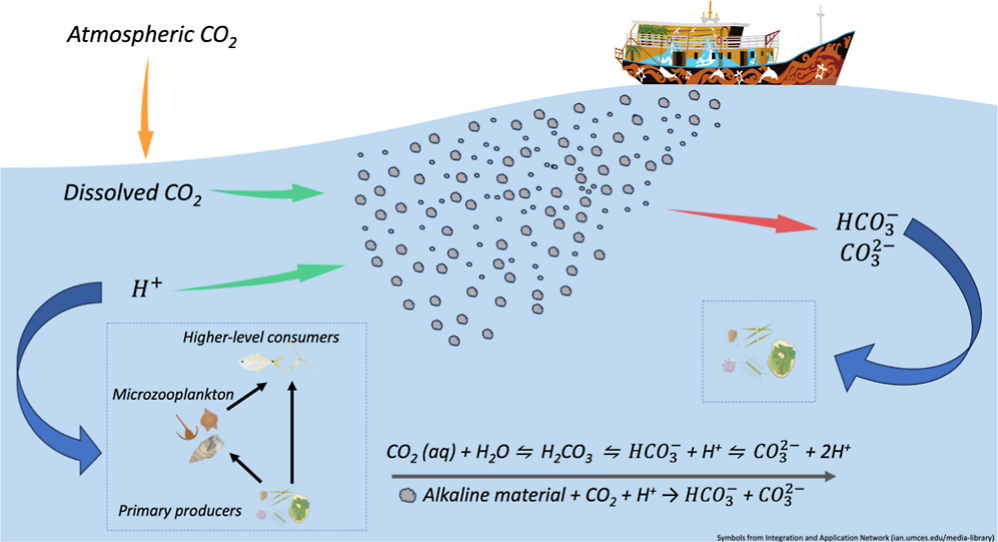

Ocean alkalinity enhancement (OAE) is currently discussed as a potential negative emission technology to sequester atmospheric carbon dioxide in seawater. Yet, its potential risks or cobenefits for marine ecosystems are still mostly unknown, thus hampering its evaluation for large-scale application. Here, we assessed the impacts OAE may have on plankton communities, focusing on phytoplankton and microzooplankton. In a mesocosm study in the oligotrophic subtropical North Atlantic, we investigated the response of a natural plankton community to CO_2_-equilibrated OAE across a gradient from ambient alkalinity (2400 μmol kg^–1^) to double (4800 μmol kg^–1^). Abundance and biomass of phytoplankton and microzooplankton were insensitive to OAE across all size classes (pico, nano and micro), nutritional modes (autotrophic, mixotrophic and heterotrophic) and taxonomic groups (cyanobacteria, diatoms, haptophytes, dinoflagellates, and ciliates). Consequently, plankton communities under OAE maintained their natural chlorophyll *a* levels, size structure, taxonomic composition and biodiversity. These findings suggest a high tolerance of phytoplankton and microzooplankton to CO_2_-equilibrated OAE in the oligotrophic ocean. However, alternative application schemes involving more drastic perturbations in water chemistry and nutrient-rich ecosystems require further investigation. Nevertheless, our study on idealized OAE will help develop an environmentally safe operating space for this climate change mitigation solution.

## Abstract 2

Studies on the ecological impacts of OAE are scarce. This study reports responses of marine oligotrophic ecosystems to OAE to provide insights into the applicability and scalability of this carbon dioxide removal technology.

## Introduction

Anthropogenetic carbon dioxide (CO_2_) emissions have led to severe climate change, pushing both natural and human systems beyond their capacity to adapt.^1^ Severer impacts on the climate, ecosystems and human society are likely if immediate actions are not taken. The delay in achieving substantial and enduring reductions in short-term CO_2_ emissions, coupled with the rising ambition of long-term climate policy goals, have propelled the concept of negative emission technologies (NETs) to the forefront of international discussions on climate policy.^2−5^ To align with the international climate goals of counteracting global warming in the 1.5 °C pathways established by the Paris Agreement, approximately 1 to 15 GtCO_2_ yr^–1^ must be captured by the end of this century.^6,7^

Ocean alkalinity enhancement (OAE) is one of the least represented technologies of CO_2_ removal from the NETs portfolio in the literature.^8^ However, if conducted at appropriate scales, OAE holds the potential to remove substantial amounts of carbon from the atmosphere.^9,10^ Many environmental, social, and ethical questions would have to be addressed before the large-scale deployment of OAE. This technology aims to enhance ocean carbon uptake by introducing alkaline materials, including electrochemically generated forms of alkalinity (in the form of hydroxide) or pulverized/dissolved alkaline minerals and industrial byproducts along the coastlines and into seawater.^11,12^ The equation of total alkalinity (TA) reads as
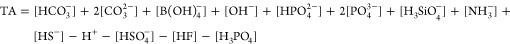
1

The enhancement in TA facilitates the conversion of CO_2_ into bicarbonate (HCO_3_^–^) and carbonate (CO_3_^2–^) ions and, consequently, reduces the partial pressure of CO_2_ (*p*CO_2_). The disparity in *p*CO_2_ between the ocean and the atmosphere could prompt the ocean to absorb additional CO_2_ from the atmosphere and decrease the outgassing of CO_2_. Thus, in addition to capturing CO_2_ from the atmosphere, OAE has the cobenefit of mitigating ocean acidification, a major and growing concern for marine ecosystems.^13^ Theoretically, OAE can be conducted in two different approaches regarding its impacts on seawater–carbonate chemistry: (I) “CO_2_-non-equilibrated”: only alkalinity is increased, while dissolved inorganic carbon (DIC) subsequently increases through CO_2_ uptake via air-sea gas exchange (until *p*CO_2_ is in equilibrium with the atmosphere again, which can take weeks to years^14^), (II) “CO_2_-equilibrated”: OAE entails adding a corresponding amount of DIC together with alkalinity so that *p*CO_2_ remains in equilibrium with the atmosphere, thereby resulting in much weaker carbonate chemistry perturbations. With respect to real application scenarios, CO_2_-nonequilibrated OAE could be conducted by adding alkaline material directly into the ocean, whereas CO_2_-equilibrated OAE would involve simultaneously adjusting *p*CO_2_ of the seawater, e.g. by preparing an alkaline solution and injecting DIC for instantaneous equilibration with atmospheric CO_2_ using special reactors (Figure S1). Both approaches provide inherent advantages and face practical constraints, including limitations in time, space, human resources, financial costs, required expertise and potential ecological disturbance.^15^ CO_2_-equilibrated OAE is expected to result in fewer side effects due to its lower carbon chemistry perturbation, compared to CO_2_-nonequilibrated OAE. While the temporal dynamics in carbonate chemistry differ according to these different approaches, the final OAE state of both approaches would be CO_2_ equilibrium between air and seawater, characterized by an increase in HCO_3_^–^, CO_3_^2–^ and pH. In real-world applications, the release of alkaline materials would be deployed at discrete locations, which could generate perturbation plumes with decreasing intensity, both spatially, from the release site toward the periphery, and temporally, as the alkalized patch gradually dilutes through mixing over time.^12,16^

These changes driven by CO_2_-equilibrated OAE in carbonate chemistry could have direct and indirect impacts on plankton communities, which form the base of ocean food webs and play a key role in the global carbon cycle. Phytoplankton exhibits poor efficiency in carbon utilization under current CO_2_ levels, and many species employ energy-intensive mechanisms to concentrate and take up HCO_3_^–^ as a substrate for photosynthesis (in addition to diffusive uptake of CO_2_).^17−21^ Phytoplankton can regulate physiological processes, e.g. cellular energy and nutritional budgets, to optimize carbon acquisition in response to dynamic growth conditions.^22−24^ Thus, it is conceivable that changes in carbonate chemistry driven by CO_2_-equilibrated OAE may favor photosynthesis (due to higher availability of HCO_3_) and/or favor calcifying organisms (due to higher pH and saturation state of calcium carbonate). Due to species-specific differences in diffusion limitation and carbon acquisition efficiency,^25,26^ OAE could theoretically yield variable impacts on the fitness of phytoplankton, thus affecting community composition and diversity. Microzooplankton, as major consumers of primary production, could be impacted indirectly through associated changes in prey availability but also directly through changes in pH affecting their physiology.^27^ This, in turn, could trigger trophic cascades affecting upper trophic levels and microbial loops.^28^

Altogether, it is presently unclear whether OAE may affect the fitness of planktonic organisms, the species diversity and size distribution within plankton communities, and ultimately, the energy transfer in the food web and fluxes in the global carbon cycle.^29−31^ Although it may seem obvious to use results from ocean acidification research to inform potential impacts of OAE (as ocean acidification changes carbonate chemistry in the opposite direction), it should be noted that possible impacts on marine ecosystems could manifest in an asymmetric way, meaning that effects of OAE cannot be deduced by just assuming opposite effects of existing ocean acidification studies.^32,33^ Thus, it is imperative to better examine the potential ecological effects of OAE in dedicated studies before considering its larger-scale application. Improved knowledge of the ecological consequence of OAE on plankton communities is indispensable to evaluate the applicability and scalability of this negative emission technique.

In this study, we present results from an in situ mesocosm experiment designed to assess how a natural plankton community responds to OAE perturbation. We simulated CO_2_-equilibrated OAE, thereby avoiding drastic shifts in carbonate chemistry. The primary objective was to evaluate the ecological risks and/or cobenefits of CO_2_-equilibrated OAE for phytoplankton and microzooplankton communities. We provide insights to develop an environmentally safe operating space for this carbon management strategy.

## Methods

### In Situ Mesocosm Experiment Design and Setup

Nine units of mesocosms were deployed at the pier of Taliarte harbor (27°59′24″ N, 15°22′8″ W), located on the east coast of Gran Canaria, Spain, from September to October 2021. Each mesocosm, consisting of a cylindrical polyurethane foil bag as well as a conical sediment trap, was installed in a floatation frame. The mesocosm tops were covered by transparent plastic roofs, preventing precipitation and bird droppings. Seawater, drawn from outside the harbor using a peristaltic pump, was evenly distributed into the mesocosms using digital flow meters, resulting in a final volume of ∼8 m^3^. Natural oligotrophic plankton communities were enclosed, while larger organisms and patchily distributed nekton were excluded through a 3 mm mesh. To maintain the characteristics of the oligotrophic system, no additional nutrients were introduced, and OAE was simulated in the mesocosms under nearly identical starting conditions.

A gradient of nine CO_2_-equilibrated OAE treatments was established by injecting HCO_3_^–^ and CO_3_^2–^ enriched seawater as alkaline feedstock on Day 4 (see eq 1), allowing the plankton communities to acclimate during the initial 3 days and establishing baseline values for the subsequent measurements. The gradient design could explore a wide spectrum of potential OAE deployment intensities to identify the threshold of OAE effects and ensure sufficient statistical power in the analysis. Additionally, the gradient approach is resilient to the loss of one or more mesocosms, a concern in in situ mesocosm experiments due to the high infrastructure costs and logistical challenges involved.^34−36^ The upper limit, corresponding to double the ambient seawater, was determined by the saturation state of calcite (Ω_Ca_), with values between 15–20 potentially resulting in secondary precipitation of calcium carbonate (CaCO_3_).^37,38^ Alkalinity-enriched water was added using a special distribution device (“spider”, Figure S2) to ensure homogeneous mixing inside the mesocosms. Regular cleaning of both the inside and outside of the mesocosms was performed to minimize fouling organism growth on the walls and maintain consistent light intensity. For a comprehensive description of the experimental design and technical details, please refer to Paul et al. (2024).^39^

### Sample Collection and Measurements

Samples were collected daily before alkalinity addition and at two-day intervals afterward using a custom-built sampler, which consisted of 2.5 m long polypropylene tubing with a valve at each end, allowing for the collection of 5 L of water evenly throughout the water column of the mesocosms (Figure S3). These samples (a total volume of 10 L for each mesocosm) were then transferred to canisters and transported in the dark to nearby laboratory facilities, where they were subsampled for every parameter.

Samples for dissolved inorganic carbon (DIC) and total alkalinity (TA) were collected directly from the sampler into 250 mL glass flasks and filtered to remove particles. TA was measured by potentiometric titration with HCl, following Chen et al. (2022).^40^ DIC was analyzed by infrared absorption (LI-COR LI-7000, AIRICA, MARIANDA). Seawater standards verified TA and DIC accuracy, with a maximum DIC variability of 10.2 μmol kg^–1^ over the first 3 days. The carbonate system variables, including CO_2_, Ω_Ar_ and pH, were calculated using K1 and K2 equilibrium constants as these align well with direct measurements of DIC and TA.^41,42^

Subsamples for nutrients (inorganic nitrate + nitrite, phosphate and silicate) were collected in acid-cleaned polycarbonate bottles at the pier, filtered (0.45 μm Sterivex, Merck), and analyzed spectrophotometrically using an Autoanalyser (QuAAtro, SEAL Analytical) with an autosampler (XY2 autosampler, SEAL Analytical) and fluorescence detector (FP-202, JASCO).

For pigment analyses, subsamples ranging between 1000 and 1500 mL were filtered through glass fiber filters (GF/F Whatman, pore size: 0.7 μm), with precautions taken to minimize exposure to light, and sequentially frozen at −20 °C until analysis. Pigments were extracted in 100% acetone and measured using a Thermo Scientific HPLC (Ultimate 3000, Thermo Scientific).^43^ In this study, the sum of chlorophyll *a* and divinyl chlorophyll *a*, which is distinctive in *Prochlorococcus* sp., was considered as total chlorophyll *a* (Chla).

In accordance with the timing of alkalinity addition and Chla dynamics, we divided the experiment into three phases. Phase 0, spanning from day 1 until day 3, covers the period before alkalinity addition. Phase I, from day 5 to day 19, incorporates the initial response of the plankton community to alkalinity addition. The observed increase in Chla, compared to that on Day 3, marks the initiation of Phase II, which encompasses the event of a longer-term response. Contributions of individual phytoplankton groups were estimated using CHEMTAX, which optimizes the initial ratios of pigment to chlorophyll *a* of phytoplankton groups for the best fit with bulk pigment concentrations.^44^ The initial pigment ratio was compiled from Higgins et al. (2011).^45^ Successive runs were performed to obtain the correct adjustment and, therefore, biomass estimate of major algal classes.

To quantify picophytoplankton (0.2–2 μm; *Synechococcus-*like cyanobacteria and picoeukaryotes) and nanophytoplankton (2–10 μm; nanoeukaryotes), we employed a Cytosense scanning flow cytometer (Cytobuoy b.v., Netherlands) with a laser excitation wavelength of 488 nm, 20 mW. The instrument recorded the pulse shapes of forward scatter (FWS), sideward scatter (SWS), as well as red, orange, and yellow fluorescence (FLR, FLO, FLY, respectively) signals for each particle. Unfixed samples were analyzed with a sheath flow rate of 60 cm^3^/min, a red fluorescence trigger (FLR 10 mV), and a 180 s acquisition time. Particle sizes were calibrated using nonfluorescent spherical beads and FWS data, and biovolumes were estimated assuming spherical shapes for all cells. Carbon content was estimated with conversion factors based on the literature: 230 fgC μm^–3^ for *Synechococcus*, 237 fgC μm^–3^ for picoeukaryotes^46^ and 220 fgC μm^–3^ for nanoeukaryotes.^47^

For the study of microplankton (size range of 10–200 μm), a volume of 250 mL of seawater was collected at four-day intervals and fixed with Lugol’s solution to achieve a final concentration of approximately 0.5%. These samples were then stored in brown glass bottles in the dark until measurement. The abundances of both microphytoplankton and microzooplankton were determined using a Zeiss Axiovert 100 microscope following the Utermöhl technique,^48^ with cells classified to the lowest identifiable taxonomic level.^49,50^ Size measurements for both groups were conducted on samples collected on days 1, 19, and 33. Measurements were performed on all species to calculate species-specific biovolumes based on their most appropriate geometry.^51^ Subsequent conversion from biovolumes to biomass was conducted following the method described by Menden–Deuer and Lessard (2000) (*C* [pg Cell^–1^] = 0.288 V^0.811^ for diatoms; *C* [pg Cell^–1^] = 0.216 V^0.939^ for other taxonomic phytoplankton groups and microzooplankton).^52^

### Data Analysis

Diversity (*H′*), species richness (*D*) and evenness (*J*) of the microplankton community (10–200 μm) were estimated based on microscopy data.

*H′* was estimated with the Shannon Weaver diversity index
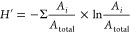
where *A*_*i*_ is the abundance of the species *i* and *A*_total_ the abundance of all individuals. Higher *H′* denotes a higher diversity.

*D* was estimated with Menhinick’s index
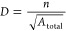
where *n* is the number of species.

*J* was estimated with Pielou evenness
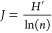


The more variation in abundances between different taxa within the community, the lower *J*.

To identify the potential ecological effects of OAE on plankton community structure, we conducted multivariate analyses with the help of the “vegan” package in R.^53,54^ The plankton community data comprised phytoplankton concentrations (μg L^–1^) derived from pigment to chlorophyll *a* ratios via HPLC and CHEMTAX, as well as microzooplankton biomass (μg mL^–1^) from microscopy.

To account for the different scales of biomass among these diverse groups, we applied data normalization using the formula
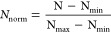
where *N*_norm_ represents the normalized value of parameter *N*. *N*_min_ and *N*_max_ refer to the lowest and highest values of parameter *N* across all mesocosms on a sampling day. These normalized values were then averaged according to treatments and over time within different phases of the experiment. This scaling standardized the data to a range between 0 and 1 while preserving overall sample variance and relative differences between mesocosms. After data normalization, nonmetric multidimensional scaling (NMDS) analysis was performed using the Bray–Curtis dissimilarity method to generate ecological distance matrices and conduct multivariate analyses. Dissimilarities between alkalinity levels were mapped for visualization. Points that were located in close proximity to one another indicate similarity.

Linear regression analysis was used to identify potential statistically significant correlations between the mean values of measurement parameters of each experimental phase and delta TA concentrations. The Mantel Test was employed to confirm whether differences in plankton community composition resulted from alkalinity addition did not occur by chance. Statistical significance was assumed for *p* < 0.05. All data analysis was performed in R environment Version 4.2.3.^54^

## Results

### Carbonate Chemistry and Nutrient Conditions

**Figure 1:**
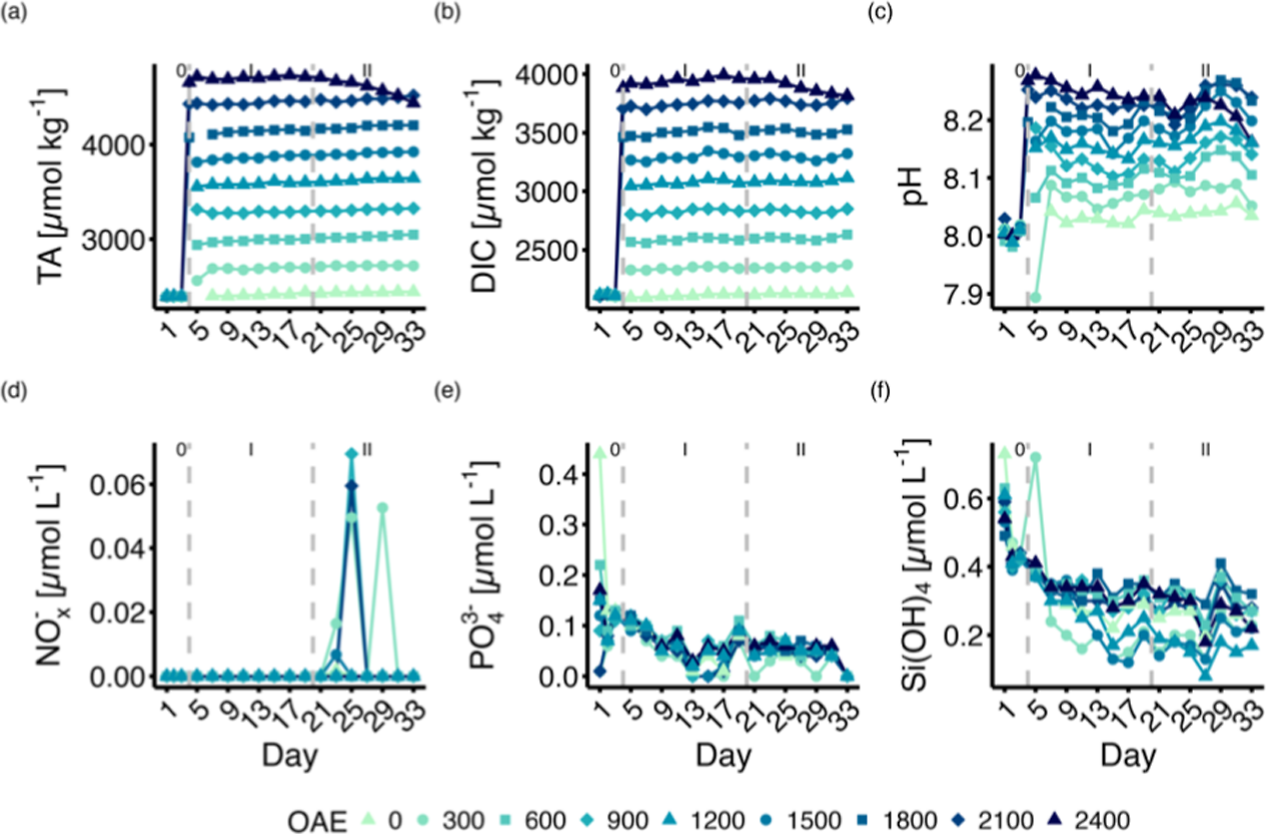
Temporal development of carbonate chemistry: (a) TA, (b) DIC and (c) pH. Temporal development of inorganic nutrients: (d) NO_*x*_^–^, (e) PO_4_^3–^ and (f) Si(OH)_4_. Dashed lines and Roman numbers indicate the different phases of the experiment.

The targeted OAE levels were successfully reached (Figure 1a–c; Table 1). From Day 21 onward, a decrease in both TA and DIC was observed under the highest OAE, with losses of ∼270 and ∼140 μmol kg^–1^, respectively, by the end of the study.

**Table 1:** Average Carbonate Chemistry System Including HCO_3_^–^, CO_3_^2–^, CO_2_, pH and Saturation State of Aragonite after Alkalinity Addition

	carbonate chemistry (μmol kg^–1^)				
ΔTA	HCO_3_^–^	CO_3_^2-^	CO_2_	pH	Ω_Ar_
0	1889.48	219.49	416.49	8.04	3.41
300	2038.0	246.20	439.0	8.04	3.99
600	2270.86	310.12	426.51	8.11	4.82
900	2456.03	361.12	428.70	8.14	5.61
1200	2659.93	412.79	437.22	8.16	6.41
1500	2814.70	468.10	434.60	8.20	7.27
1800	2969.01	528.76	428.15	8.22	8.21
2100	3160.70	578.60	442.50	8.23	8.99
2400	3298.68	608.19	459.37	8.24	9.45

Throughout the study, nitrate and nitrite concentrations (combined as NO_*x*_^–^) remained consistently low, frequently falling below the detection threshold of the analytical methods used (Figure 1d). Inorganic phosphate (PO_4_^3–^) decreased by 0.1 μmol L^–1^ during the first 13 days, after which it stabilized at consistently low values (Figure 1e). Silicate concentrations (Si(OH)_4_) were initially measured between 0.42 and 0.44 μmol L^–1^ (Figure 1f), with a reduction observed, ranging from 0.07 to 0.22 μmol L^–1^ between Day 3 and Day 19, followed by negligible changes thereafter.

### Phytoplankton Community Composition and Size Structure

**Figure 2:**
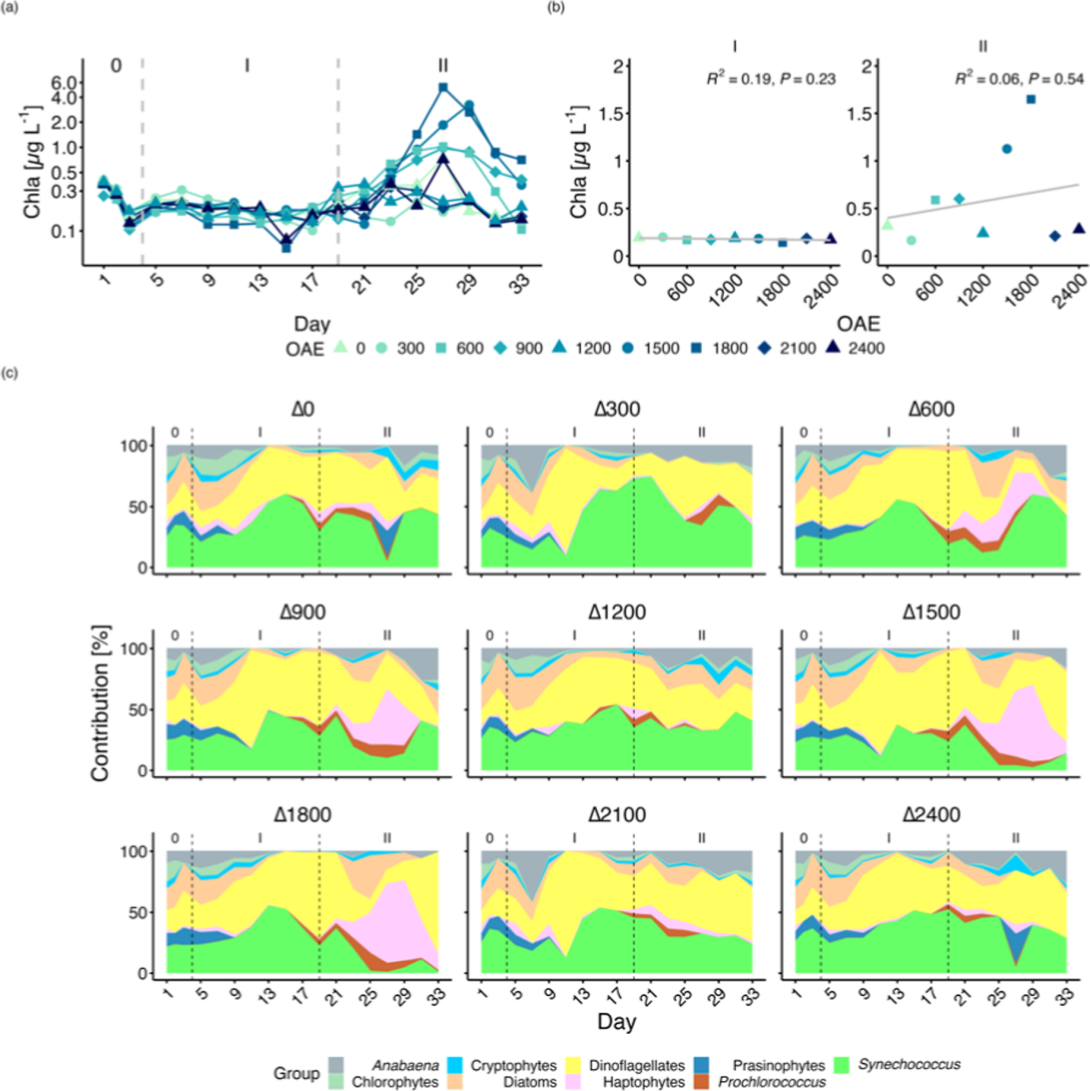
Temporal development and linear regression analysis on the average over time of Chla (a). Note that the *y*-axis of Chla is on a logarithmic scale. Relative chlorophyll *a* contribution of each phytoplankton group to Chla (b). Dashed lines and Roman numbers indicate the different phases of the experiment.

In phases 0 and I, Chla concentrations showed no significant changes relative to TA (Figure 2a). High *p*-values indicated no significant relationship between OAE and Chla. However, in Phase II, Chla concentrations unexpectedly increased in treatments ΔTA600, ΔTA900, ΔTA1500 and ΔTA1800, while no bloom was observed in ΔTA1200, ΔTA2100 and ΔTA2400. Linear regression analysis conducted on Chla and each taxonomic group indicated no significant relationship between OAE and any of the plankton community variables (Figure 2a, Table S1). Thus, the observed differences in Chla and taxonomic groups among mesocosms are not attributable to OAE. This implies that these blooms were random and unrelated to OAE.

Microscopic observation identified the blooming nanophytoplankton species (generally ∼6 μm) as a motile, noncalcifying haptophyte, *Braarudosphaera bigelowii* (formerly *Chrysochromulina parkeae*), characterized by its nitrogen-fixing endosymbiont UCYN-A.^55^ The study location falls within the distribution of UCYN-A nitrogenase gene sequences, according to GenBank.^56^ This species cannot utilize most inorganic nitrogen sources that larger phytoplankton generally have a higher affinity for. Instead, it relies on nitrogen generated by its endosymbiont under organic nitrogen-rich environments, contributing to its outcompetition during Phase II.^39,57,58^

No increase in planktonic calcifiers was observed either immediately after the addition of alkalinity or during the second phase. During Phase 0 and I, *Synechococcus* contributed the largest proportion to Chla in all mesocosms, with diatoms and mixotrophic dinoflagellates contributing to a lesser extent (Figure 2b). In contrast, in Phase II, Chla was dominated by the noncalcifying haptophytes in ΔTA900, ΔTA1500 and ΔTA1800 treatments (Figure 2b).

Community compositions remained similar following the addition in Phase I. Phase II revealed increased dissimilarity, with mesocosms experiencing blooms clustering in the lower-left region of the NMDS plot, mainly driven by haptophytes and *Synechococcus* (Figure S4). However, linear regression analysis and Mantel analysis confirmed that TA did not influence the community compositions either in Phase I or II (Table S2).

**Figure 3:**
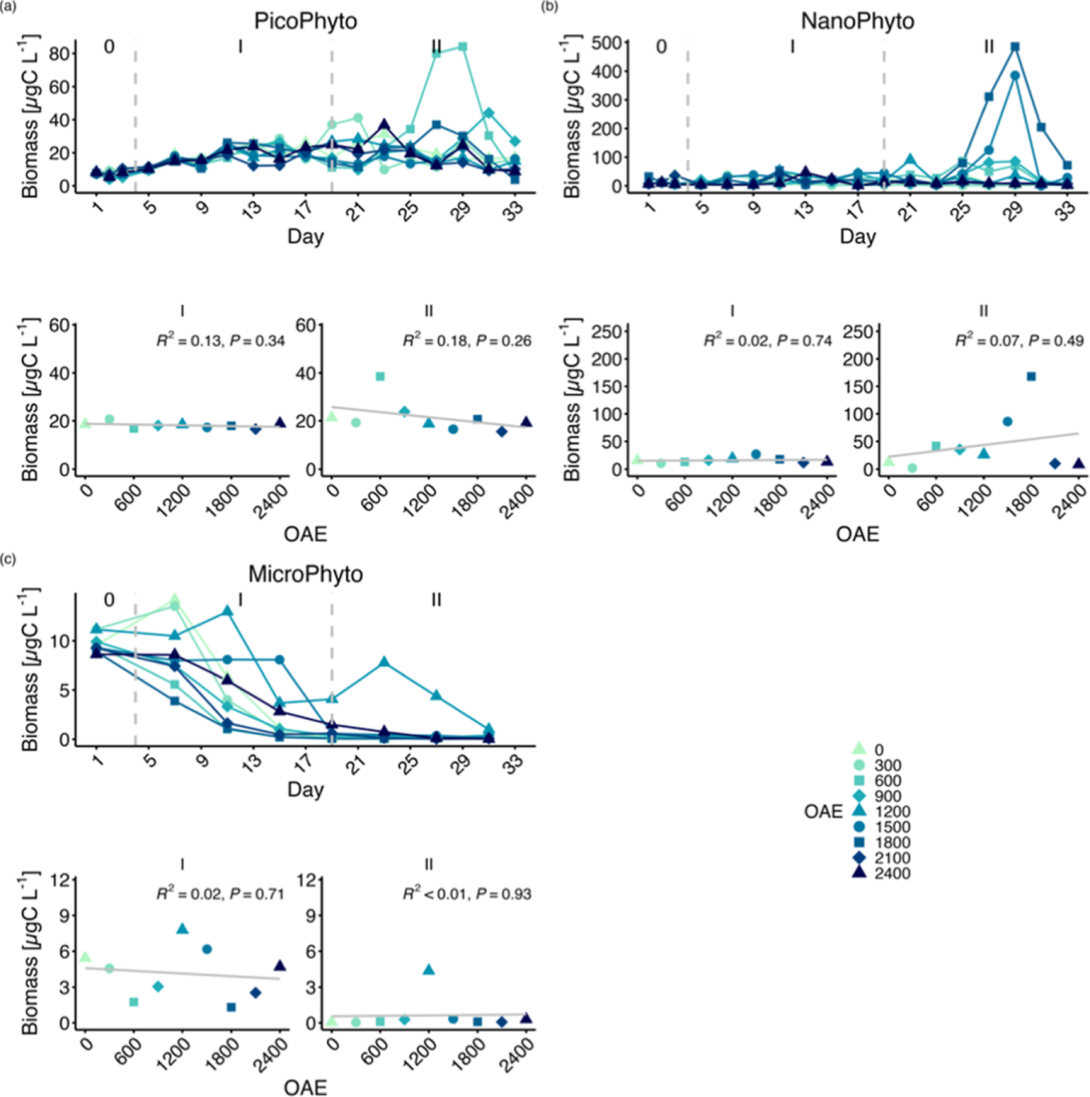
Carbon biomass across all size structures of phytoplankton: (a) picophytoplankton (size <2 μm), (b) nanophytoplankton (2–10 μm), (c) microphytoplankton (≥10 μm). Top panels show the temporal development of each mesocosm and bottom panel regression on the average over time. Roman numbers indicate the different phases of the experiment.

Irrespective of TA, the biomass of each size group of phytoplankton within mesocosms changed with time (Figure 3). Initially, the biomass of picophytoplankton exhibited an increasing trend and remained relatively stable during Phase II (Figure 3a). Nanophytoplankton showed a stable trend in biomass in Phase I, with notable increases in ΔTA600, ΔTA900, ΔTA1500 and ΔTA1800 treatments during Phase II (Figure 3b). Large autotrophs, in general, declined with the gradual reduction in P and Si (Figures 3c; S5). The phytoplankton of each group displayed no sensitivity to changes in TA (Figure 3; Table S3), thus leaving the size structure of the phytoplankton community unaffected by OAE.

### Microzooplankton Community Composition, Nutrition Mode and Size Structure

**Figure 4:**
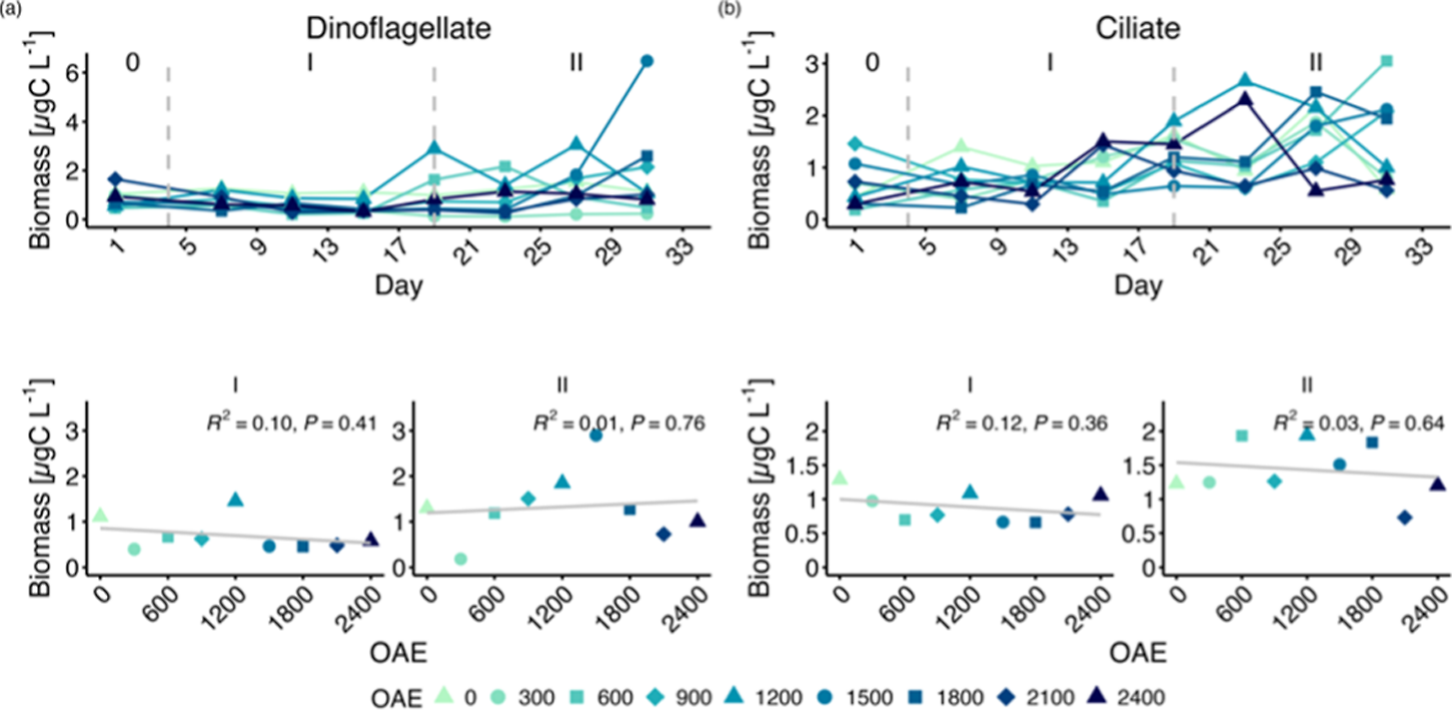
Carbon biomass across dominant microzooplankton groups: (a) dinoflagellate and (b) ciliate. Top panels show the temporal development of each mesocosm and bottom panel regression on the average over time. Roman numbers indicate the different phases of the experiment.

**Figure 5:**
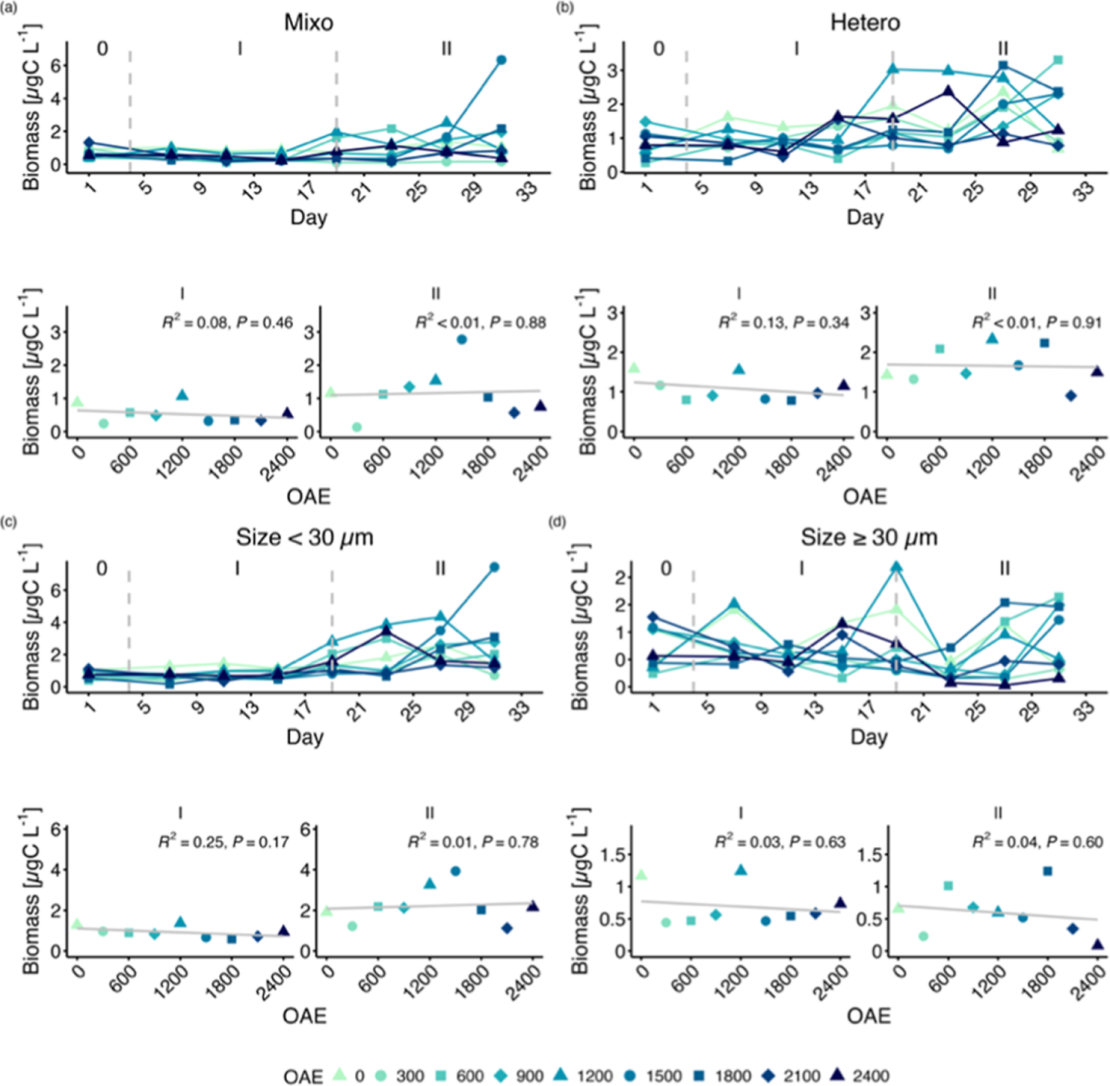
Carbon biomass across all modes of nutrition and size structures of microzooplankton: (a) mixotrophic microzooplankton, (b) heterotrophic microzooplankton, (c) small microzooplankton (size <30 μm) and (d) large microzooplankton (≥30 μm). Top panels show the temporal development of each mesocosm and bottom panel regression on the average over time. Roman numbers indicate the different phases of the experiment.

There were some temporal changes in the biomass of microzooplankton groups dominated by mixo/heterotrophic dinoflagellates and ciliates as identified by microscopy analysis (Figures 4; S6). The carbon biomass of the microzooplankton community was initially low and increasingly dominated by mixotrophic/heterotrophic dinoflagellates, including *Gymnodinium* sp*.,* and heterotrophic ciliates, including *Lohmanniella* sp*.* during succession (Figure S7). However, these changes occurred irrespective of TA levels (Figure 4; Table S4). The rise in dinoflagellates and ciliates led to an increase in both mixotrophic and heterotrophic microzooplankton, contributing to a greater abundance of small-sized microzooplankton. The biomass of both microzooplankton size groups and nutritional modes were unaffected by OAE (Figure 5).

### Plankton Diversity

**Figure 6:**
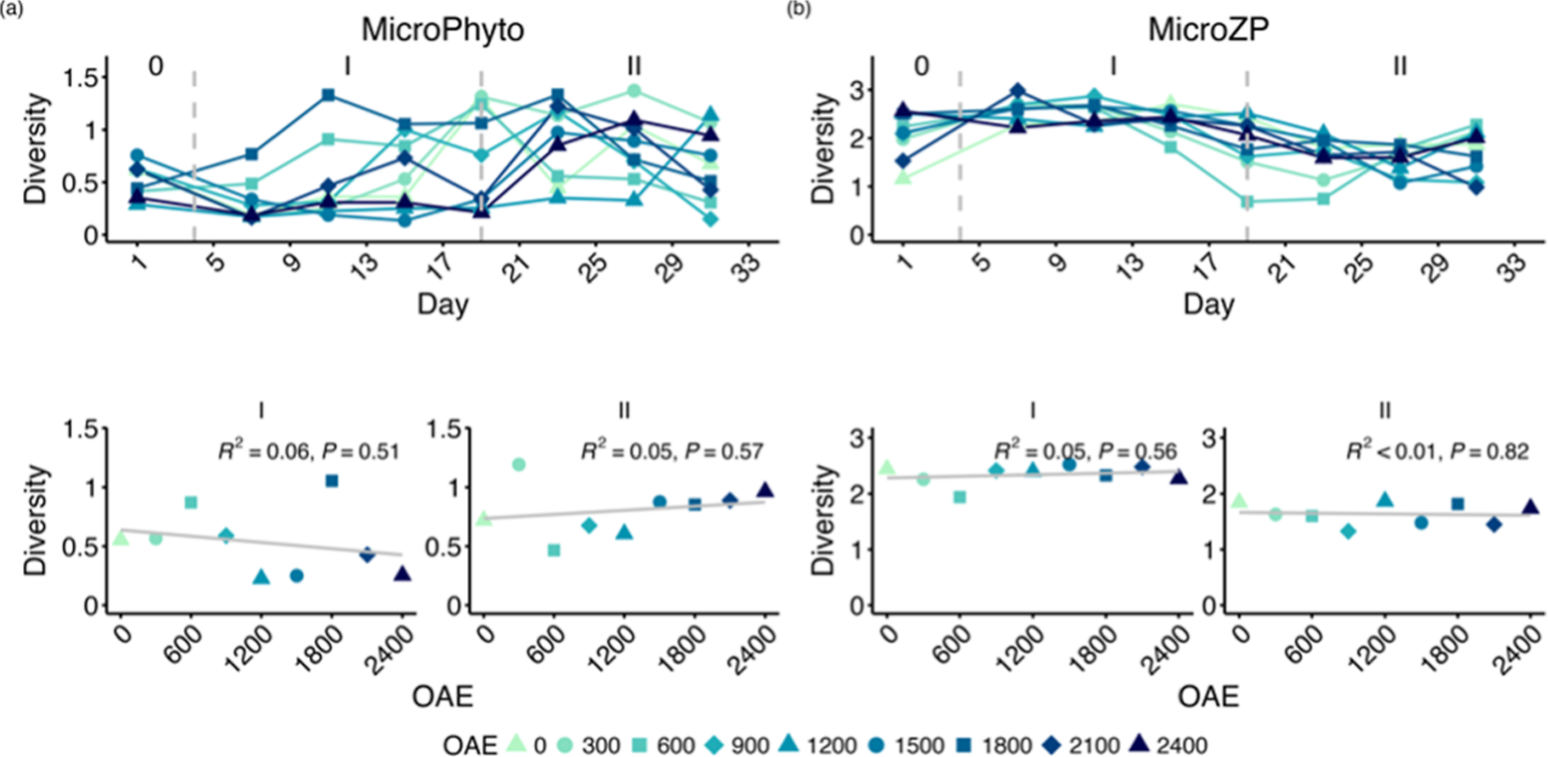
Shannon-Weaver diversity of (a) microphytoplankton and (b) microzooplankton community. Top panels show the temporal development of each mesocosm and bottom panel regression on the average over time. Roman numbers indicate the different phases of the experiment.

OAE exhibited no significant impact on the diversity of either microphytoplankton or microzooplankton communities (based on species level, Figure 6). In Phase 0, the phytoplankton community was dominated by the diatom *Leptocylindrus minimus*, contributing to low species diversity. The diminishment of this species, attributed to the depletion of nutrients, yielded a slight increase in phytoplankton diversity over time. In contrast, the microzooplankton community was dominated by increasing dinoflagellates, e.g. *Gymnodinium* sp*.,* and ciliates, e.g. *Lohmanniella* sp*.*, leading to a subsequent decrease in overall microzooplankton diversity. Species richness and evenness were slightly affected by the succession in the plankton community. However, neither species richness nor evenness changed depending on the TA level (Figure S8).

## Discussion

Our study addresses the influence of CO_2_-equilibrated OAE on pelagic plankton communities in the subtropical North Atlantic. Both phytoplankton and microzooplankton communities exhibited resilience to OAE disturbance, indicating that this carbon removal technology may be applied with minimal ecological side effects in oligotrophic areas.

### No TA Effects Detectable on the Stability of the Phytoplankton Community

Due to the CO_2_-equilibrated OAE applied in this study, the concentration of HCO_3_^–^ was elevated while that of CO_2_ was maintained stable. Thus, the only way phytoplankton carbon acquisition could theoretically be affected would be by the increase in HCO_3_^–^. The higher availability of HCO_3_^–^ could reduce the energy required for carbon-concentrating mechanisms, leading to energy savings that could manifest as an increase in growth rate.^19^ However, we detected no discernible response of phytoplankton to OAE. Most likely, the prevailing carbonate chemistry conditions (abundant ambient CO_2_ for diffusive uptake) resulted in low selection for HCO_3_^–^, as the process of HCO_3_^–^ entry into the cell is associated with higher energy expenditure.^19^ CO_2_ was the preferred carbon substrate, and opting for CO_2_ uptake over HCO_3_^–^ pathways could provide energy savings.^59−62^ In addition, saturation thresholds of dominant species of representative phytoplankton functional groups—the diatom *Skeletonema costatum*, the flagellate *Phaeocystis globosa*, and the coccolithophore *Emiliania huxleyi*—were reported in earlier studies to fall far below the current ocean HCO_3_^–^ levels.^59,63^ This makes it unlikely that the OAE-driven increase in HCO_3_^–^ would be harnessed by phytoplankton, particularly in the presence of abundant CO_2_ in the mesocosms.

Besides the changes in HCO_3_^–^, OAE in our experiment also elevated pH by up to 0.23 units. Consistent declines in growth rate and photosynthesis in phytoplankton were observed in previous studies when pH levels were higher than 8.8.^64^ In comparison, the rather slight changes in pH during our OAE application are on the same levels as typical pH variations in most oceanic regions, i.e. within the range phytoplankton could readily tolerate.^65^ In addition, studies dissecting the independent role of high pH and CO_2_ suggest that high pH per se is unlikely to impact the fitness of phytoplankton. Generally, the sensitivity of phytoplankton to high pH likely differs from the sensitivity to low pH, which stems from the increase in H^+^ in the stroma of the chloroplast and leads to reduced CO_2_ fixation efficiency.^66−68^ Instead, in the case of high pH, the limited availability of CO_2_ that usually coincides with the increase in pH in natural systems is the actual mechanism causing the reduction in photosynthesis, carbon fixation rate and growth.^33,69,70^ However, our CO_2_-equilibrated OAE scenario differs from such pH and CO_2_ dynamics—here the increase in pH occurs at constant CO_2_ concentrations, thus making it much less likely to impact phytoplankton growth and community structure.

Changes in the trophic modes, size distribution and diversity of microzooplankton may disrupt marine biogeochemical cycles of bioactive elements within the food web, given their crucial ecological functions.^28^ In our study, the impacts of OAE on microzooplankton, mainly comprising mixo- and heterotrophic dinoflagellates as well as ciliates, were not detectable. Regarding the potential direct effects of carbonate chemistry, most microzooplankton species exhibit tolerance to high pH, generally surpassing the upper pH level tested in our study.^71^ Furthermore, no OAE-related changes occurred in phytoplankton (Figure 2; Table S1) and mesozooplankton,^72^ which could theoretically affect microzooplankton.^73−75^ Hence, both potential drivers for indirect OAE effects on microzooplankton (food availability and grazing pressure) were unaffected by the OAE treatment.

A further potential explanation for the absence of an effect of OAE in our study could be the low nutrient conditions in our study region. Throughout the experimental period, inorganic nutrients were predominantly below the detection threshold and thus, within the typical range of local observations for oligotrophic conditions.^76,77^ Thus, it is conceivable that the effects of OAE did not manifest in microphytoplankton due to these nutrient-depleted conditions.^78−80^ Insights from preceding ocean acidification mesocosm experiments conducted in the same region suggest that the effects of ocean acidification were generally minimal under oligotrophic conditions, with effects largely emerging after the addition of nutrients, i.e. during bloom and postbloom conditions.^81^ Thus, low nutrient concentrations may, to some extent, have constrained the potential for the emergence of OAE effects: oligotrophic conditions generally favor small phytoplankton species and usually inhibit the growth of larger primary producers due to differences in nutrient utilization efficiency.^82,83^ These differences in cell size (and surface-to-volume ratio) also affect carbon utilization strategies and their efficiency, resulting in larger species being more limited by diffusive CO_2_ uptake and the necessity to take up HCO_3_^–^ via carbon-concentrating mechanisms. Accordingly, it is possible that phytoplankton communities dominated by larger species are more responsive to OAE-related changes in carbonate chemistry. Thus, further studies are required to assess the responses of different phytoplankton communities in different nutritional states to OAE.

### Implications for the Assessment of Ocean Alkalinity Enhancement

Our results suggest that plankton communities are resilient to CO_2_-equilibrated OAE under oligotrophic conditions. The abundance of pico- and nanophytoplankton remained stable and even increased, suggesting the nutrient concentration could meet the nutrient demand of pico- and nanophytoplankton. The observed decrease in microphytoplankton in oligotrophic conditions is a common phenomenon under such conditions. The response of microphytoplankton in the short term phase (Phase I) suggests that OAE posed no disturbance to the microphytoplankton community. Overall, our findings suggest that phytoplankton remained active, and the impacts of OAE were negligible. Additional data from this study also found that neither biogeochemical processes, such as carbon export nor primary production, were affected by OAE.^84,85^ It should be emphasized that the range of alkalinity conditions in our study was wide. Secondary CaCO_3_ precipitation was observed in the high OAE treatments, indicative of potential temporal extremes that should be avoided during episodic and long-term scenarios of alkalization.^38,86^ As carbonate chemistry conditions at the coast of the Canary Islands are typically stable (an increase of 0.85 μmol kg^–1^ yr^–1^ in DIC), local phytoplankton communities can be assumed to be relatively sensitive to perturbations.^81,87^ Thus, the absence of OAE effects on plankton communities during our study gives confidence that this carbon removal technology could generally be implemented with minimal negative ecological side effects under CO_2_-equilibrated OAE conditions. Our findings are in line with the few existing studies, suggesting that OAE may not cause a major disturbance to primary production processes.^88,89^ In contrast, a prior microcosm study showed that elevated alkalinity moderately but significantly impacted the characteristics of bloom and associated succession of the phytoplankton community.^90^ However, rather than OAE, the shift was conceivably attributed to the disparity in initial community structures as identified within the nanophytoplankton that dominate phytoplankton biomass.^90,91^ The presence of impurities from alkaline materials may emerge as a concern regarding the impacts on the marine ecosystem. The dissolution products, e.g. iron, silicate and phosphate, could provide essential nutrients that benefit phytoplankton.^92−94^ Trace metals, such as nickel, could enhance the growth of cyanobacteria; however, excess concentrations could be toxic to marine organisms.^92,95^ It should be noted that the effects of coreleased products are both biome- and concentration-dependent. Thus, the limited evidence so far suggests that CO_2_-equilibrated OAE is likely to have no or moderate impacts on plankton communities while caution should be taken with the dissolution products that could be coreleased during OAE.

Such plankton communities dominated by small species in our study are representative of the majority of open ocean areas, which are oligotrophic and with a size distribution of organisms skewed heavily toward picophytoplankton.^29,96−98^ If our results hold true on a larger temporal and spatial scale, the indication that the deployment of OAE bears a low risk of perturbing the plankton community suggests the feasibility of OAE in oligotrophic areas. Nevertheless, we have to acknowledge the uncertainties of our limited study duration, as well as regional variations in plankton community composition and environmental conditions. Additionally, ecosystems in certain regions, e.g. seasonal seas and upwelling areas, might be less limited by nutrients. Once released from the constraints of nutrients, responses of the plankton community might emerge or be amplified during the process of OAE (analogous to findings from ocean acidification studies). Thus, further studies in different biomes are essential.

Another factor is the application mode of OAE. Though bearing a minimal risk of posing carbonate chemistry disturbance to the plankton community, the CO_2_-equilibrated OAE is characterized by the high capital and operating cost, e.g. for reactors to dissolve alkaline substances and capture CO_2_, compared to those of the CO_2_-nonequilibrated OAE.^16^ For technical and economic considerations, OAE may be implemented in scenarios where equilibrium is disrupted, leading to more pronounced carbonate chemistry perturbations, e.g. a reduction in CO_2_ and a sharp increase in pH. In such scenarios, primary production might be impeded, as CO_2_ serves as the primary substrate for most phytoplankton species. This could potentially favor species with strategies for HCO_3_^–^ utilization that then have a selective advantage over those reliant exclusively on CO_2_ or highly sensitive to the availability of CO_2_ as *p*CO_2_ decreases and becomes limited.^20,99^ Consequently, variations in phytoplankton carbon acquisition efficiency may trigger shifts in community composition. The associated increase in pH is unlikely to directly impede the growth of phytoplankton but could indirectly affect the plankton community by decreasing the availability of trace metals, which are essential for phytoplankton growth.^100,101^ Severe increase in pH in unequilibrated scenarios could also affect zooplankton fitness, which could consequently affect the phytoplankton community and upper trophic levels through top-down and bottom-up control, respectively.^71,99,102^ It should be noted that CO_2_-nonequilibrated OAE is more prone to alkalinity loss, hindering the Monitoring, Reporting and Verification of OAE.^103^ Thus, the threshold for CO_2_-nonequilibrated OAE should be set lower than that for CO_2_-equilibrated OAE, limiting the efficiency of this CO_2_ removal approach. Overall, these considerations suggest that the response of plankton communities to OAE could differ in CO_2_-nonequilibrated conditions. More comprehensive studies focusing on influences caused by CO_2_-nonequilibrated OAE and efforts to mitigate these changes, e.g. deployment of alkalinity enhancement in zones with high mixing dynamics, are suggested to better understand and mitigate these detrimental consequences.

Our in situ study, being the first conducted at a large-volume scale and with a subtropical plankton community, carries important implications for future study of OAE. First, our results suggest the potential applicability and practicality of this technology in CO_2_ mitigation without causing substantial ecological disruptions if OAE is applied under the right boundary conditions. Second, our results imply that the low-nutrient conditions prevailing in large parts of the ocean could be leveraged during the deployment of equilibrated alkalinity to minimize potential risks to local ecosystems. Third, our findings provide insights into future environmentally safe operating spaces for OAE as a promising solution for climate change mitigation. Nevertheless, OAE responses may differ under other environmental conditions and ecological contexts, and further studies are imperative to enable a comprehensive assessment of the large-scale applicability of OAE.

## Data Availability

The raw data supporting the conclusions of this article will be made available by the authors, without undue reservation. The data will be submitted to Pangaea, https://www.pangaea.de/.

## Supporting Information Available

Diagram for CO_2_-equilibrated and nonequilibrated approaches; Distribution device (spider) used to facilitate the uniform enhancement of alkalinity during addition; Experimental timeline for the mesocosm; Linear regression analysis of taxonomic groups of phytoplankton and dominant microzooplankton; NMDS analysis of the plankton community; Linear regression and Mantel test statistics of the relationship between ecological distance and environmental distance; Carbon biomass across dominant microphytoplankton species; Linear regression analysis of dominant microphytoplankton species; Contribution of dominant microzooplankton genus relative to the total carbon biomass; Carbon biomass across dominant microzooplankton genera; Linear regression analysis of dominant microzooplankton genera; Richness and evenness of microphytoplankton and microzooplankton; Initial pigment ratios of CHEMTAX analysis for mesocosms without bloom; Initial pigment ratios of CHEMTAX analysis for mesocosms with bloom (PDF)
