# Supplementary material for: Resilience of Phytoplankton and Microzooplankton Communities under Ocean Alkalinity Enhancement in the Oligotrophic Ocean
Source: Environ Sci Technol. 2024 Nov 11;58(47):20918–30. doi: 10.1021/acs.est.4c09838 (PMC11603778; doi:10.1021/acs.est.4c09838)
Supplement: Supplementary file 1 — es4c09838_si_001.pdf [file es4c09838_si_001.pdf]

Resilience of phytoplankton and microzooplankton communities under ocean alkalinity enhancement in the oligotrophic ocean: Supporting information

Xiaoke Xin [†, *], Silvan Urs Goldenberg [†], Jan Taucher [†], Annegret Stuhr [†], Javier Arístegui [‡] and Ulf Riebesell [†]

[†] GEOMAR Helmholtz Centre for Ocean Research, Wischhofstraße 1-3, 24148 Kiel, Germany

[‡] Instituto de Oceanografía y Cambio Global (IOCAG), Universidad de Las Palmas de Gran Canaria, Parque Científico Tecnológico Marino de Taliarte, s/n, 35214 Telde, Las Palmas, Spain

[*] Correspondence to: Xiaoke Xin (xxin@geomar.de)

Number of pages: 8
Number of figures: 8
Number of tables : 6

Figure S1 : Diagram for CO_2-equilibrated and CO_2-non-equilibrated OAE approaches.
Figure S2 : Distribution device (spider) used to facilitate the uniform enhancement of alkalinity during addition.
Figure S3 : Experimental timeline for the mesocosm.
Table S1 : Linear regression analysis of taxonomic groups of phytoplankton and dominant microzooplankton.
Figure S4 : NMDS analysis of the plankton community.
Table S2 : Linear regression and Mantel test statistics of the relationship between ecological distance and environmental distance.
Figure S5 : Carbon biomass across dominant microphytoplankton species.
Table S3 : Linear regression analysis of dominant microphytoplankton species.
Figure S6 : Contribution of dominant microzooplankton genus relative to the total carbon biomass.
Figure S7 : Carbon biomass across dominant microzooplankton genera.
Table S4 : Linear regression analysis of dominant microzooplankton genera.
Figure S8 : Richness and evenness of microphytoplankton and microzooplankton.
Table S5 : Initial pigment ratios of CHEMTAX analysis for mesocosms without bloom.
Table S6 : Initial pigment ratios of CHEMTAX analysis for mesocosms with bloom.

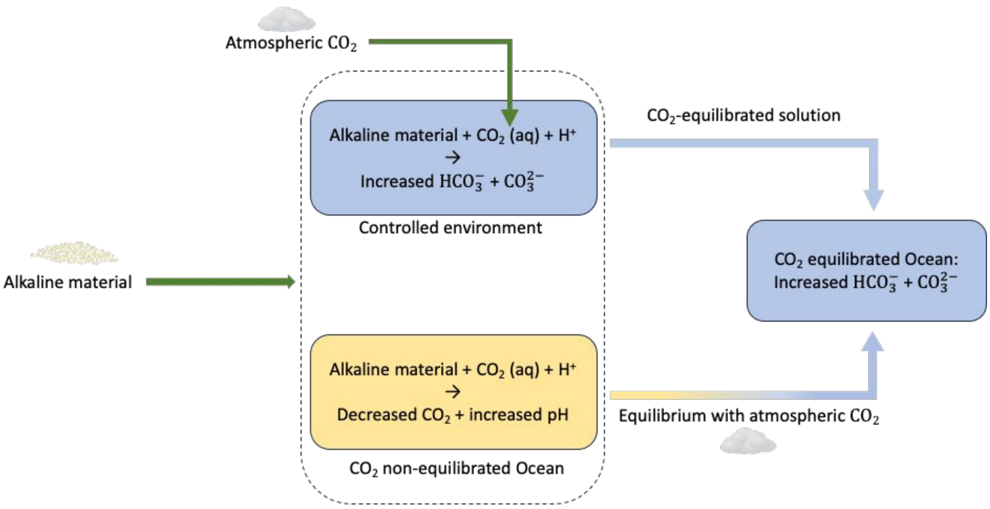

Figure S1. CO_2-equilibrated and CO_2-non-equilibrated approaches for ocean alkalinity enhancement. In CO_2-equilibrated OAE approach, CO_2 is absorbed and the equilibrium with atmosphere is restored in controlled environments, e.g. industrial reactors and natural basins, before alkaline solution is introduced into the ocean. In CO_2-non-equilibrated OAE approach, the alkaline material is directly introduced into the ocean where CO_2 is consumed initially and the discrepancy between the ocean and atmosphere drives the ocean to absorb atmospheric CO_2 and to restore equilibrium. Symbols from the Integration and Application Network (ian.umces.edu/media-library).

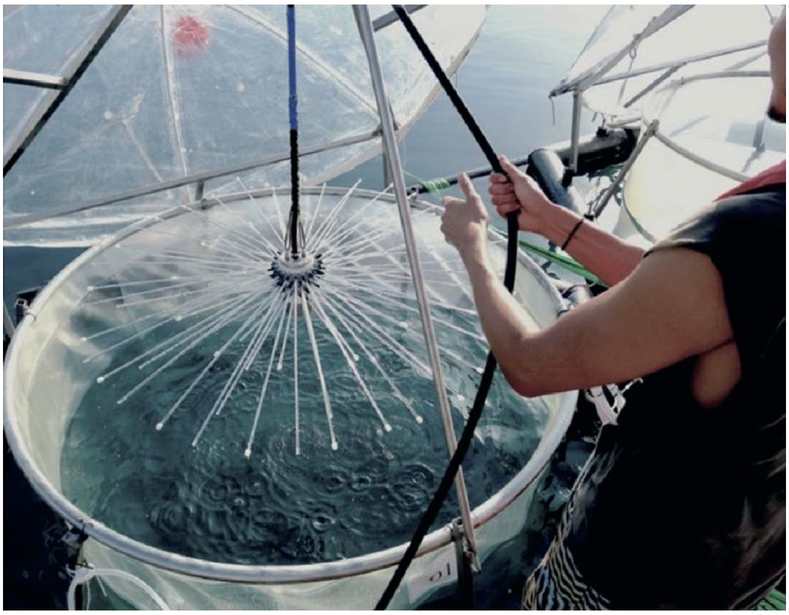

Figure S2. Spider used to facilitate the uniform enhancement of alkalinity during addition. By moving the device up and down the water column at a consistent speed during the injection process, alkalinity is evenly dispersed throughout the water. Photo source: Ulf Riebesell.

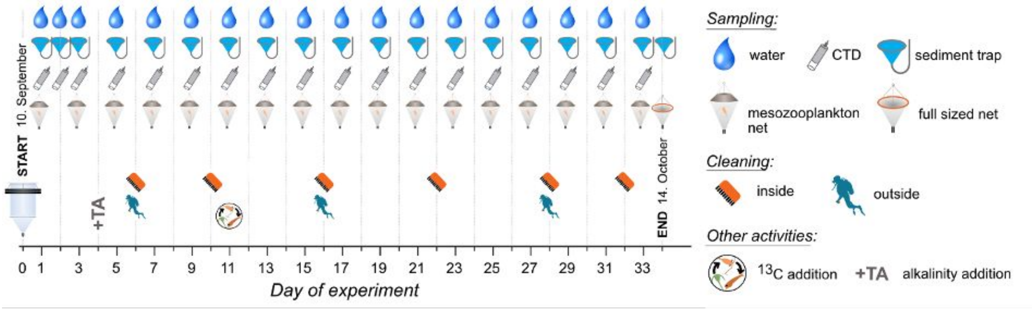

Figure S3. Experimental timeline for the mesocosm indicating sampling, cleaning and other activities. For a comprehensive description of the experimental design and technical details, please refer to Paul et al. (2024) [1].

Table S1. Linear regression analysis of taxonomic groups of phytoplankton and dominant microzooplankton.

Data source	Taxonomic group	Phase I			Phase II		
		R^2	F	*p*	R^2	F	*p*
CHEMTAX	*Synechococcus*	-0.02	0.84	0.39	0.10	1.89	0.21
	Diatoms	-0.07	0.45	0.52	-0.09	0.32	0.59
	Haptophytes	0.10	1.86	0.22	-0.05	0.62	0.46
	Cryptophytes	0.06	1.49	0.26	-0.08	0.37	0.56
	Chlorophytes	0.07	1.6	0.25	-0.16	0.18	0.69
	Prasinophytes	-0.11	0.19	0.67	-0.14	0.03	0.87
	Dinoflagellates (Auto&Mixo)	-0.13	0.07	0.81	-0.06	0.57	0.47
	Anabaena	-0.12	0.12	0.74	-0.14	0.05	0.84
	Prochlorococcus	-0.09	0.35	0.58	-0.09	0.35	0.56
Microscopy	Dinoflagellates (Hetero)	-0.02	0.85	0.39	0.13	2.23	0.18
	Ciliates	-0.01	0.95	0.36	-0.11	0.24	0.64
	Unidentified microzooplankton	-0.14	0.01	0.99	-0.13	0.09	0.77

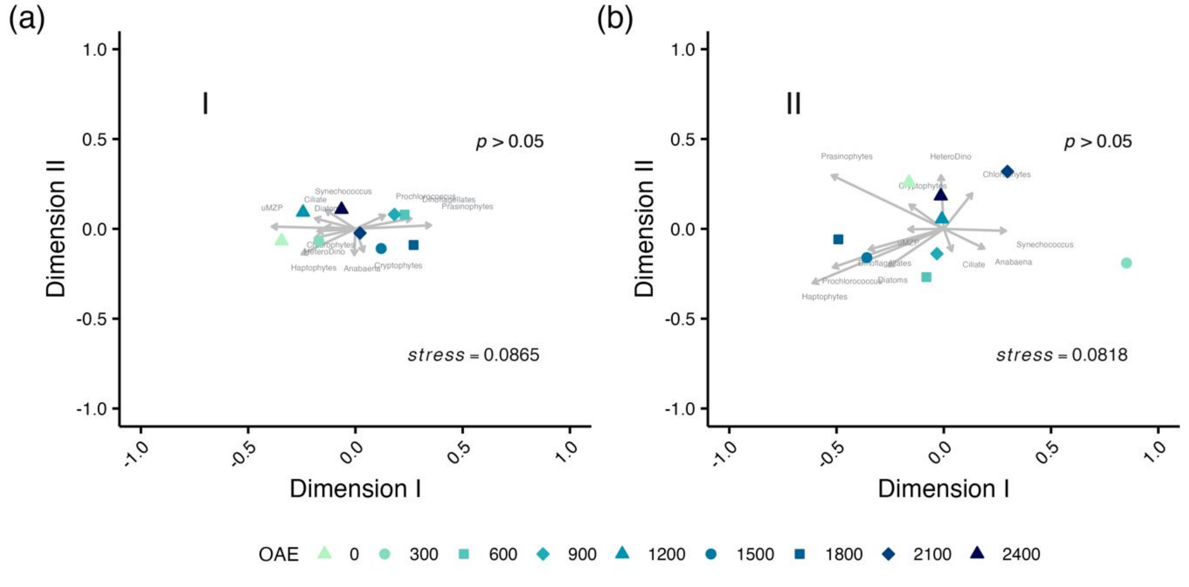

Figure S4. NMDS analysis of the plankton community in phase I (a), and phase II (b). Stress values lower than 0.2 indicate an ideal representation in reduced dimensions.

Table S2. Linear regression and Mantel test statistics of the relationship between ecological distance and environmental distance. Significant dissimilarities among plankton communities were present with $p < 0.05$.

Phase	Linear Regression			Mantel Test	
	R^2	F	*p*	r	*p*
I	-0.04	0.70	0.43	-0.09	0.66
II	-0.10	0.26	0.63	0.06	0.35

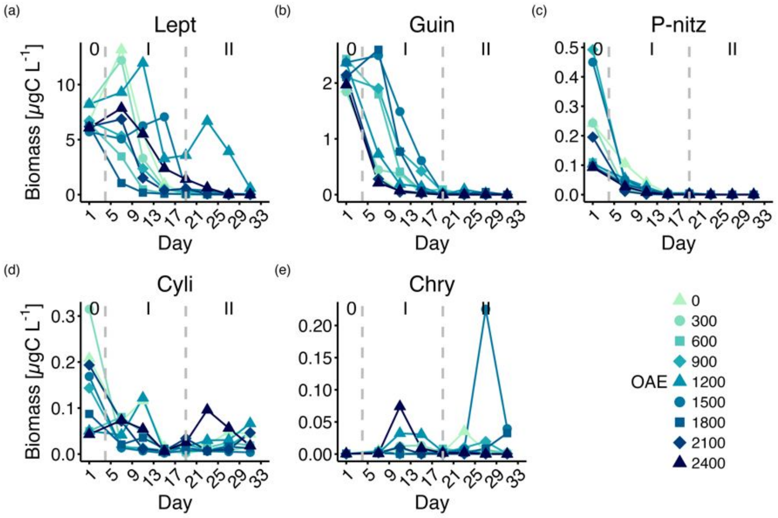

Figure S5: Carbon biomass across dominant microphytoplankton species: (a) *Leptocylindrus minimus*; (b) *Guinardia striata*; (c) *Pseudo-nitzschia cf. delicatissima*; (d) *Cylindrotheca closterium*; (e) *Chrysochromulina lanceolata* (>10 µm). Top panels show temporal development of each mesocosm and bottom panel regression on the average over time. Roman numbers indicate the different phases of the experiment.

Table S3. Linear regression analysis of dominant microphytoplankton species.

Group	Taxonomic group	Phase I			Phase II		
		R^2	F	*p*	R^2	F	*p*
Phytoplankton	*Leptocylindrus minimus*	-0.12	0.133	0.73	-0.14	0.01	0.93
	Guinardia striata	-0.14	0.01	0.91	-0.11	0.23	0.65
	Pseudo-nitzschia cf. delicatissima	0.22	3.12	0.12	0.18	2.76	0.14
	Cylindrotheca closterium	-0.13	0.06	0.81	-0.10	0.29	0.61
	Chrysochromulina lanceolata (>10 µm)	-0.03	0.76	0.42	-0.14	0.03	0.86

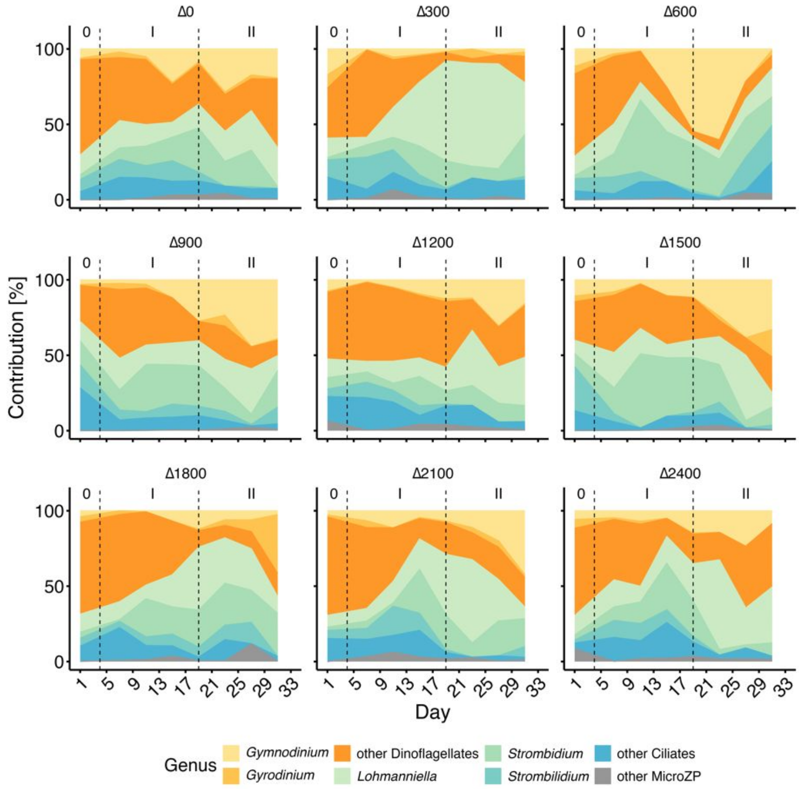

Figure S6. Contribution of dominant microzooplankton genus relative to the total carbon biomass. Less dominant genera are pooled as other MicroZP. Dashed lines and Roman numbers indicate the different phases of the experiment.

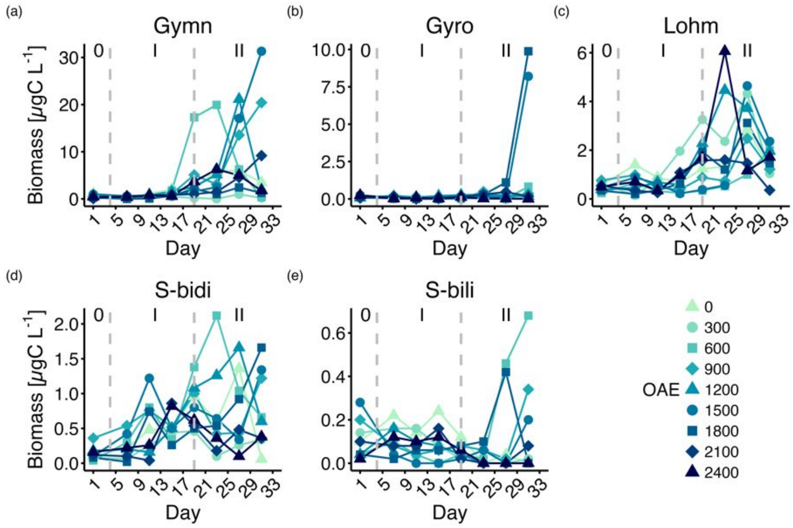

Figure S7: Carbon biomass across dominant microzooplankton genera: (a) *Gymnodinium spp.*; (b) *Gyrodinium spp.*; (c) *Lohmanniella sp.*; (d) *Strombidium spp.*; (e) *Strombilidium spp.*. Top panels show temporal development of each mesocosm and bottom panel regression on the average over time. Roman numbers indicate the different phases of the experiment.

Table S4. Linear regression analysis of dominant microzooplankton genera.

Group	Taxonomic group	Phase I			Phase II		
		R^2	F	*p*	R^2	F	*p*
Microzooplankton: Dinoflagellates	*Gymnodinium spp.*	0.03	1.1	0.31	-0.14	0.04	0.86
	Gyrodinium spp.	0.06	1.52	0.26	-0.05	0.64	0.45
Microzooplankton: Ciliates	*Lohmanniella sp.*	-0.06	0.58	0.47	-0.11	0.20	0.67
	Strombidium spp.	-0.08	0.39	055	-0.11	0.19	0.67
	Strombilidium spp.	0.04	1.35	0.28	-0.11	0.21	0.66

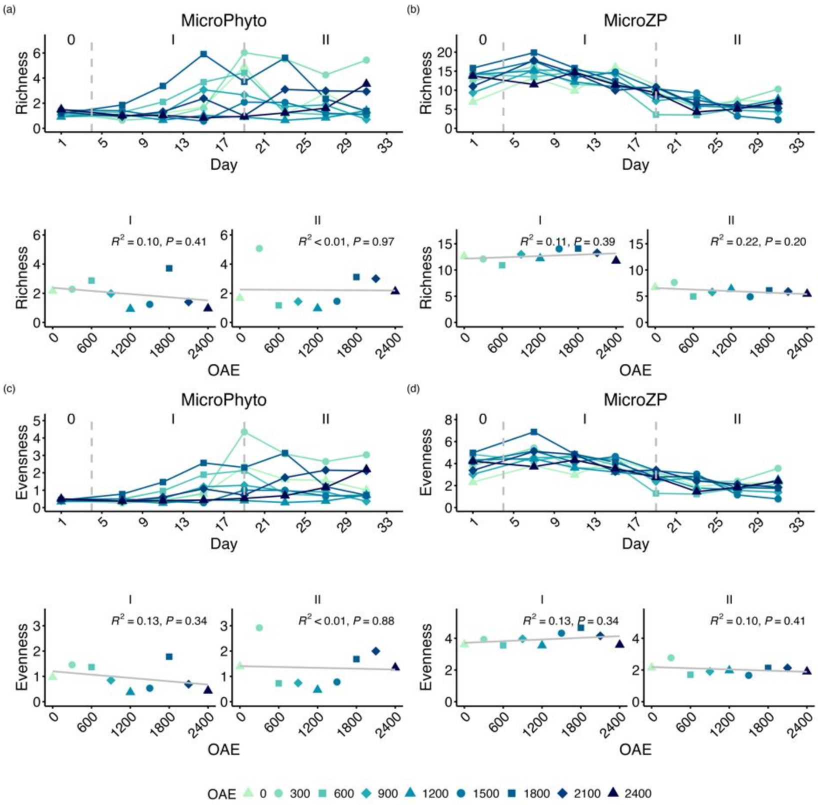

Figure S8. Richness of (a) microphytoplankton and (b) microzooplankton community. Evenness of (c) microphytoplankton and (d) microzooplankton community. Top panels show temporal development of each mesocosm and bottom panel regression on the average over time. Roman numbers indicate the different phases of the experiment.

Table S5. Initial pigment ratios of CHEMTAX analysis for mesocosms without bloom. A total of four runs was performed.

Pigment Selection	1	1	1	1	1	1	1	1	1	1	1	1	1	1	1	1	1	1	1
Class / Pigment	Chlc3	Chlc2	Peri	19-But	Fuco	Neox	Pras	Vio	19-Hex	Asta	Diadino	Myxo	Allo	Diatox	Zea	Chlb	Chlc2-mgdg	divinyl-Chla	Chla
Prasinophytes	0	0	0	0	0	0.0647	0.2506	0.0542	0	0	0	0	0	0	0.0587	0.7032	0	0	1
Chlorophytes	0	0	0	0	0	0.066	0	0.049	0	0.0123	0	0	0	0	0.032	0.315	0	0	1
Cryptophytes	0	0.104	0	0	0	0	0	0	0	0	0	0	0.253	0	0	0	0	0	1
Diatoms	0	0.179	0	0	0.775	0	0	0.001	0	0	0.163	0	0	0.028	0.003	0	0	0	1
Diatoms2	0.083	0.284	0	0	0.998	0	0	0	0	0	0	0	0	0	0	0	0	0	1
Dinoflagellates1	0	0.245	0.804	0	0	0	0	0	0	0	0.177	0	0	0.076	0	0	0	0	1
Dinoflagellates2	0.205	0.125	0	0.079	0.219	0	0	0	0.135	0	0.079	0	0	0	0	0	0	0	1
Haptophytes	0.214	0.094	0	0	0.608	0	0	0	0.044	0	0	0	0	0	0	0	0.031	0	1
Synechococcus	0	0	0	0	0	0	0	0	0	0	0	0	0	0	0.45	0	0	0	1
Anabaena	0	0	0	0	0	0	0	0	0	0	0	0.189	0	0	0.004	0	0	0	1
Prochlorococcus	0	0	0	0	0	0	0	0	0	0	0	0	0	0	0.389	0	0	1	1

Table S6. Initial pigment ratios of CHEMTAX analysis for mesocosms with bloom. A total of four runs was performed.

Pigment Selection	1	1	1	1	1	1	1	1	1	1	1	1	1	1	1	1	1	1	1
Class / Pigment	Chlc3	Chlc2	Peri	19-But	Fuco	Neo	Pras	Vio	19-Hex	Asta	Diadino	Myxo	Allo	Diatox	Zea	Chlb	chlc2-mgdg	divinyl-Chla	Chla
Prasinophytes	0	0	0	0	0	0.0647	0.2506	0.0542	0	0	0	0	0	0	0.0587	0.7032	0	0	1
Chlorophytes	0	0	0	0	0	0.066	0	0.049	0	0.0123	0	0	0	0	0.032	0.315	0	0	1
Cryptophytes	0	0.104	0	0	0	0	0	0	0	0	0	0	0.253	0	0	0	0	0	1
Diatoms	0	0	0	0	0.76	0	0	0	0	0	0	0	0	0	0	0	0	0	1
Dinoflagellates1	0	0.245	0.804	0	0	0	0	0	0	0	0.177	0	0	0.076	0	0	0	0	1
Dinoflagellates2	0.205	0.125	0	0.079	0.219	0	0	0	0.135	0	0.079	0	0	0	0	0	0	0	1
Haptophytes	0.214	0.094	0	0	0.608	0	0	0	0.044	0	0	0	0	0	0	0	0.031	0	1
Synechococcus	0	0	0	0	0	0	0	0	0	0	0	0	0	0	0.45	0	0	0	1
Anabaena	0	0	0	0	0	0	0	0	0	0	0	0.189	0	0	0.004	0	0	0	1
Prochlorococcus	0	0	0	0	0	0	0	0	0	0	0	0	0	0	0.389	0	0	1	1

Reference

1. Paul, A. J.; Haunost, M.; Goldenberg, S. U.; Hartmann, J.; Sánchez, N.; Schneider, J.; Suitner, N.; Riebesell, U. Ocean alkalinity enhancement in an open ocean ecosystem: Biogeochemical responses and carbon storage durability. *EGUsphere.* **2024**.
